# Identification and Characterization of HAESA-Like Genes Involved in the Fruitlet Abscission in Litchi

**DOI:** 10.3390/ijms20235945

**Published:** 2019-11-26

**Authors:** Fei Wang, Zhihui Zheng, Ye Yuan, Jianguo Li, Minglei Zhao

**Affiliations:** 1 State Key Laboratory for Conservation and Utilization of Subtropical, Agro-Bioresources, China Litchi Research Center, South China Agricultural University, Guangzhou 510642, China; wangfeilfq@163.com (F.W.); zzh18826239036@163.com (Z.Z.); yuanye96210@163.com (Y.Y.); 2 Guangdong Litchi Engineering Research Center, College of Horticulture, South China Agricultural University, Guangzhou 510642, China

**Keywords:** litchi, fruitlet abscission, LcHSL2, floral organ abscission, AZ

## Abstract

Regulation of abscission is an important agricultural concern since precocious abscission can reduce crop yield. INFLORESCENCE DEFICIENT IN ABSCISSION (IDA) peptide and its receptors the HAESA (HAE) and HAESA-like2 (HSL2) kinases have been revealed to be core components controlling floral organ abscission in the model plant Arabidopsis. However, it is still unclear whether the homologs of IDA-HAE/HSL2 in non-model plants are correlated to abscission. Previously, we found LcIDL1, a homolog of IDA from litchi, has a similar role to AtIDA in control of floral organ abscission in Arabidopsis. Here, we further isolated an HAESA-like homolog, *LcHSL2*, which is likely involved in the fruitlet abscission in litchi. Ectopic expression of *LcHSL2* in wild type Arabidopsis has no effect on the floral organ abscission. However, its presence in the *hae hsl2* mutant background completely rescued the floral organ abscission deficiency. LcHSL2 is localized in the cell membrane and the *LcHSL2* gene is expressed at the pedicel abscission zone (AZ) of litchi and floral AZ of Arabidopsis. Real-time PCR analysis showed that the expression level of *LcHSL2* was increased during ethephon-induced fruitlet abscission in litchi. Taken together, our findings suggest that HSL2 homologs have functional conservation in Arabidopsis and litchi, and LcHSL2 might play a critical role in regulation of fruitlet abscission in litchi.

## 1. Introduction

Abscission in general is an important process in plants to shed unwanted or infected organs in response to internal or external cues [1]. However, untimely abscission of flowers or fruits is a challenge for farmers since it can cause severe losses in crop yield. Therefore, it is of great significance to understand the mechanism underlying the regulation of abscission. The abscission process in plants takes place at the cell files in the specialized abscission zone (AZ) between the organ to be shed and the main plant body. Generally, the AZ will go through three sequential developmental stages when the abscission process is activated: (i) Acquisition of competence to respond to abscission signals; (ii) cell wall loosening and expansion followed by organ separation; and (iii) transdifferentiation of the retained portion of the AZ to generate a protective layer [2,3,4]. For years, a core signaling pathway has been defined to regulate the floral organ abscission in model plant Arabidopsis [5,6,7,8,9].

In Arabidopsis, INFLORESCENCE DEFICIENT IN ABSCISSION (IDA), a small secreted peptide, plays an essential role in the regulation of abscission. Mutation in *IDA* causes the failure of floral organ abscission to take place, whereas overexpression of *IDA* results in obvious precocious abscission events [10,11]. HAESA (HAE) and HAESA-like2 (HSL2), a pair of closely related leucine-rich repeat receptor-like kinases (LRR-RLKs), function redundantly to positively regulate the floral organ abscission process since the *hae hsl2* double mutants were, like *ida*, totally deficient in abscission [5,12]. Further genetic investigation demonstrates that the role of IDA in the regulation of floral organ abscission is dependent on its receptors HAE/HSL2 [13]. Upon activation of the HAE/HSL2 by IDA, a mitogen-activated protein (MAP) kinase cascade consisting of the MAPK kinase 4 (MKK4)/MKK5 and the MAPK 3 (MPK3)/MPK6, is turned on [5], leading to the suppression of KNOTTED1-LIKE HOMEOBOX (KNOX) transcription factors, ultimately resulting in the induction of genes encoding cell wall remodeling enzymes [6]. Recently, it was shown that four somatic embryogenesis receptor kinase (SERK) family RLKs serve as co-receptors of HAE/HSL2 and form a complex with HAE/HSL2 upon binding of IDA to the receptor kinases, thus regulating the initiation of the floral organ abscission [9,14]. In contrast to many other species, Arabidopsis usually does not abscise cauline leaves, whole flowers, fruit, or leaves [15]. Orthologs of IDA-HAE/HSL2 have been identified and found to be expressed in the AZs of flowers, leaves, and fruits in all orders of flowering plants, suggesting that the IDA-HAE/HSL2 signaling module can induce the abscission processes in species besides Arabidopsis [16,17]. To date, a few cases validated the hypothesized conserved function of IDA-HAE/HSL2 across organ and species by complementation of the Arabidopsis *ida* mutant. For example, CitIDA3, a closest homolog of AtIDA in citrus, is suggested to be correlated to the process of fruit abscission since ectopic expression of *CitIDA3* caused precocious abscission in wild type Arabidopsis, and was sufficient to rescue the abscission defect when expressed in the *ida* mutant background [18]. In addition, IDA peptides enhanced the leaf abscission in *Populus* and ripe fruit abscission in oil palm, which provides additional evidence for the conservation of the IDA–HAE–HSL2 pathway in widely different abscission contexts [17]. However, the proof of conserved function of IDA-HAE/HSL2 in regulation of abscission is still lacking, particularly in fruit crops.

Litchi (*Litchi chinensis* Sonn.) is an important tropical and subtropical fruit crop that is widespread and cultivated in Southeast Asia. There are three to four waves of physiological fruit abscission throughout litchi fruit development, causing low yield and heavy economic loss [19,20]. Thus, it is of interest to identify key components that are involved in regulation of the fruit abscission. Previously, we characterized and identified LcIDL1, an IDA homolog from litchi, which functioned similarly to AtIDA in the control of floral abscission in Arabidopsis [21]. Here, we further isolated LcHSL2—a homolog of HAE/HSL2 in litchi—and found that it was involved in the fruitlet abscission in litchi and played a role in regulating the floral organ abscission in Arabidopsis.

## 2. Results

### 2.1. Identification of HAE/HSL2 Homologs in Litchi

**Figure 1:**
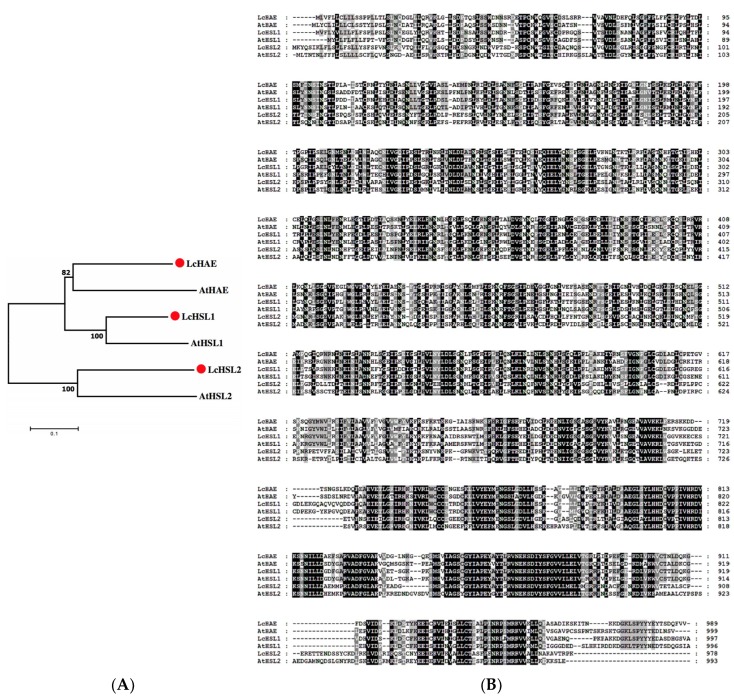
Sequence and phylogenetic analysis of LcHSLs protein. (**A**) Phylogenetic relationships of LcHSLs with Arabidopsis AtHAE, AtHSL1, and AtHSL2. LcHAE (ID number: LITCHI029130.m1), LcHSL1 (ID number: LITCHI029130.m1), and LcHSL2 (ID number: LITCHI007137.m1) are indicated by red circles. The maximum likelihood phylogenetic tree was created using MEGA program (version 5.0). (**B**) Multiple alignment of LcHAE, LcHSL1, and LcHSL2 with AtHAE, AtHSL1, and AtHSL2.

To identify the litchi HAE/HSL2 homologs, TBLASTN searches against the litchi genome (http://111.230.180.7:81/index.php) were performed at TBtools [22] using the amino acid sequences of the Arabidopsis HAE (AT4G28490), HSL1 (AT1G28440), and HSL2 (AT5G65710). As a result, three *HAE/HSL2*-like genes in the litchi genome were identified (File S1). They were named LcHAE, LcHSL1, and LcHSL2 based on their phylogenetic relationship with Arabidopsis HAE/HSL2 (Figure 1A). Protein sequence alignment showed that the amino acid similarity was 60.56% between LcHAE and AtHAE, 72.46% between LcHSL1 and AtHSL1, and 58.16% between LcHSL2 and AtHSL2 (Figure 1B). We thus hypothesized that LcHAE, LcHSL1, and LcHSL2 might have similar functions to Arabidopsis HAE/HSL2 in the regulation of abscission.

### 2.2. Ectopic Expression of LcHSL2 Complements the Abscission Deficiency of the hae hsl2 Mutant

**Figure 2:**
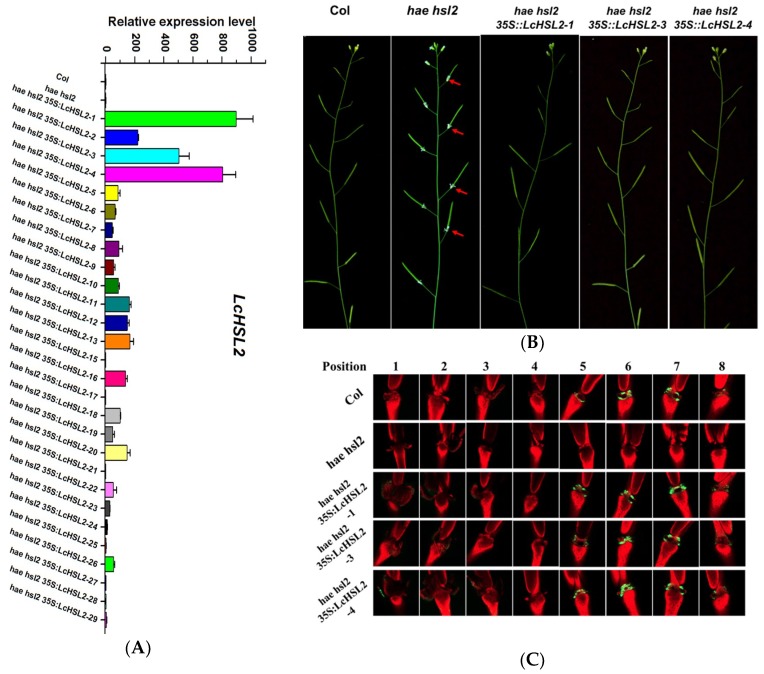
Ectopic expression of *LcHSL2* in Arabidopsis *hae hsl2* mutants rescues the floral organ abscission deficiency. (**A**) Expression levels of *LcHSL2* in transgenic lines. (**B**) Comparison of the inflorescence of *hae hsl2* and *hse hsl2 35S:LcHSL2-1*, *hse hsl2 35S:LcHSL2-3*, and *hse hsl2 35S:LcHSL2-4.* Red arrow heads indicate attached floral organs. (**C**) BCECF fluorescence micrographs of floral organ AZ of Col, *hae hsl2*, and *hse hsl2 35S:LcHSL2* transgenic lines. Inflorescences were sampled separately, incubated in BCECF solution, and examined by a confocal laser scanning microscope. The microscopic fluorescence images represent merged images of BCECF fluorescence (green) with chlorophyll auto fluorescence (red) images. The images presented for each plant and positions are representative images out of three to four replicates.

To test whether LcHAE, LcHSL1, and LcHSL2 have functions in the regulation of abscission, wild type Arabidopsis plants were transformed with a construct driving *LcHAE*, *LcHSL1*, and *LcHSL2*, respectively, by the strong constitutive cauliflower mosaic virus 35S promoter. More than 20 transgenic lines expressing each gene were generated. However, there were no visible phenotypic changes observed when compared to wild type Arabidopsis. Next, we investigated whether LcHAE, LcHSL1, and LcHSL2 had a conserved function to HAE/HSL2 in regulation of floral abscission in Arabidopsis. The *hae hsl2* mutant plants were transformed with each construct as mentioned above. We also generated more than 20 transgenic lines expressing each gene. For all the transgenic lines expressing *LcHAE* or *LcHSL1* in *hae hsl2* background, the floral organs remained attached, which was the same as that in the *hae hsl2* mutants. Interestingly, we found that 20 out of 29 T1 transgenic lines expressing *LcHSL2* showed wild type abscission patterns (three representative transgenic lines were shown here, Figure 2A,B). Previous studies have demonstrated that organ abscission is closely associated with an increase in cytosolic pH in AZ cells, which can be easily detected by BCECF-AM staining [23]. Therefore, we performed BCECF assays using three representative lines, *hae hsl2 35S:LcHSL2-1*, *hae hsl2 35S:LcHSL2-3*, and *hae hsl2 35S:LcHSL2-4*, which displayed a relatively higher expression level of *LcHSL2* in *hae hsl2* background (Figure 2A). In wild-type Col, the BCECF signals in the floral AZ appeared starting from position five and went through position eight, while the BCECF signals disappeared in the floral AZ of *hae hsl2* mutants, which is consistent with floral organ abscission deficiency of *hae hsl2*. When *LcHSL2* was expressed in *hae hsl2* mutants, the BCECF signals appeared again and showed a similar pattern to that in Col (Figure 2C). These findings indicate that the presence of *LcHSL2* was sufficient to induce the floral organ abscission in *hae hsl2* mutant plants. Thus, LcHSL2 was selected for further analysis.

### 2.3. LcHSL2 Is Localized in the Plasma Membrane and Expressed at the Floral AZ of Arabidopsis

**Figure 3:**
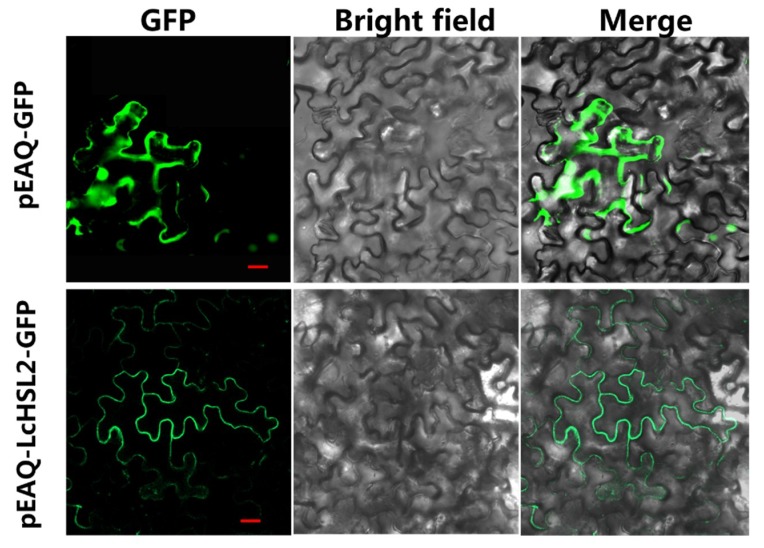
Subcellular localization assay of LcHSL2 in epidermal cells of tobacco leaves. GFP signals were observed with a fluorescence microscope after 48 h of infiltration. Bars = 25 μm.

To investigate the subcellular localization of LcHSL2, the full-length coding sequence of *LcHSL2* fused with green fluorescent protein (GFP) was transiently expressed in tobacco leaf epidermal cells. Fluorescence of fused GFP was predominantly observed in the cell membrane, and GFP signals of free control (pEAQ-GFP) were uniformly distributed throughout the whole cell (Figure 3). This indicated that LcHSL2 functions in the plasma membrane, which is consistent with its role as a receptor-like kinase.

**Figure 4:**
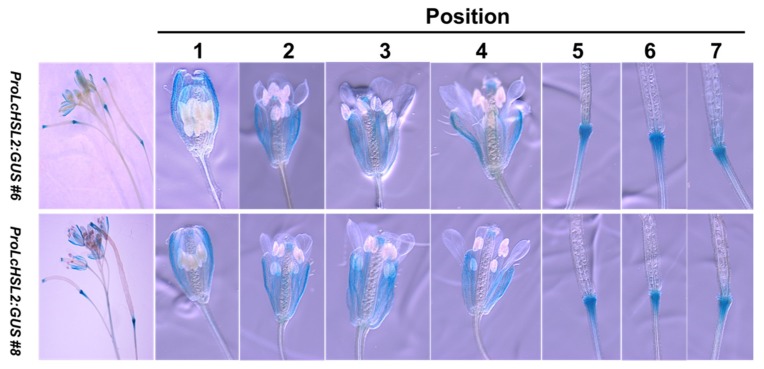
*ProLcHSL2:GUS* expression in Arabidopsis floral AZ. The numbers indicate flower position along the inflorescence. Position numbers were counted from the first flower with visible white petals at the top of the inflorescence.

In addition, we investigated the spatial and temporal activity of the *LcHSL2* promoter in Arabidopsis. A construct carrying a GUS reporter driven by the *LcHSL2* promoter was transformed into Arabidopsis. We had two transgenic lines with different expression levels of *GUS* (Figure 4). The *GUS* expression driven by the *LcHSL2* promoter was predominately localized at the floral AZ, and the GUS signals in the floral AZ appeared starting from position five (Figure 4), which is consistent with its function in the control of floral organ abscission in Arabidopsis.

### 2.4. LcHSL2 Is Expressed at the Fruit AZ and Its Expression Level Is Increased During the Fruitlet Abscission in Litchi

**Figure 5:**
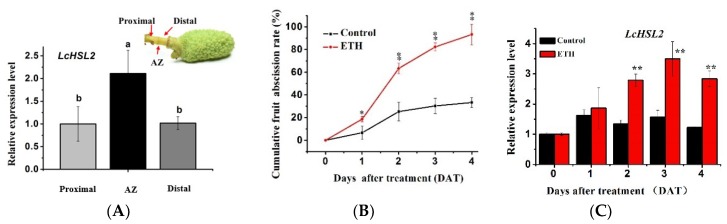
*LcHSL2* is expressed in the fruit AZ and is increased during the ETH-induced fruitlet abscission in litchi. (**A**) *LcHSL2* is predominantly expressed in the fruit AZ of litchi. Top right corner shows the fruit abscission zone (AZ), distal, and proximal regions of the litchi fruit peduncle. Different letters indicate significant differences as determined using Ducan’s multiple range test (*p* < 0.05). (**B**) Ethephon (ETH)-induced fruitlet abscission in litchi. The results are the means of three biological replicates. Error bars represent ± SE. Asterisks indicate a significant difference (Student’s *t*-test: *p* < 0.05 indicated by *; *p* < 0.01 indicated by **). (**C**) *LcHSL2* expression is increased following the ETH-induced fruitlet abscission. The *y*-axis represents fold-change in expression relative to the control at 0 day, which was set to 1. Data represent the average of three biological replicates with three technical replicates each. Error bars represent ± SE. Asterisks indicate a significant difference (Student’s *t*-test: *p* < 0.01 indicated by **).

To test the involvement of LcHSL2 in the fruitlet abscission of litchi, we examined the expression profile of *LcHSL2* in three regions of the pedicel: The fruit AZ, the distal region (between the fruitlet and the fruit AZ), and the proximal region (Figure 5A). *LcHSL2* was expressed significantly higher in the fruit AZ than those in the adjacent tissues (Figure 5A).

To gain a better understanding of the involvement of *LcHSL2* in fruitlet abscission in litchi, we examined the expression pattern of *LcHSL2* during the litchi fruitlet abscission induced by ethephon application (ETH). As shown in Figure 5B, fruitlets dropped starting from the third day after ETH treatments, the cumulative fruit abscission rate was up to 93.33% at the fifth day after ETH treatment, whereas only 33.33% of fruitlets dropped at fifth day in control (Figure 5B). qRT-PCR analysis showed that the expression level of *LcHSL2* in the pedicel AZ was significantly induced by ETH treatment, with about 2.07-fold, 2.22-fold, and 2.31-fold higher in ETH-treated AZ tissues than that in control at third, fourth, and fifth day, respectively (Figure 5C). This suggests that *LcHSL2* is associated with ethephon-induced fruitlet abscission in litchi.

## 3. Discussion

Unexpected fruit abscission is a severe problem for the litchi industry. In general, litchi trees will produce hundreds of female flowers per inflorescence, but less than 5% of the initial female flowers can develop into mature fruits [20,24]. The excessive abscission of flowers/fruitlets is one of the main factors causing universal low productivity in litchi [19,20]. Therefore, new litchi genotypes with a low rate of fruit abscission behavior derived from genetic engineering (transgenic or CRISPR gene editing technologies) would be favorable. For this, identifying the key components that are involved in regulation of fruit abscission in litchi is our priority.

In Arabidopsis, the IDA-HAE/HSL2 module was primarily found to initiate a signaling pathway in control of floral organ abscission [5,6,10,11,12]. This signaling module was also revealed to play an essential role in control of cell separation during lateral root emergence and root cap sloughing [25,26,27]. *GmIDL2a* and *GmIDL4a*, two homologs of IDA in soybean, could promote the cell separation during lateral root emergence through regulating the cell wall remodeling gene expression [28]. These findings suggest that the IDA-HAE/HSL2 signaling module can control cell separation events other than just floral organ abscission. Previously, we identified a homolog of IDA, LcIDL1, which is associated with the fruitlet abscission in litchi and has a similar function to Arabidopsis IDA in regulation of floral organ abscission [21]. In this study, we further revealed that homologs of HAE/HSL2 also exist in the litchi genome, and one member LcHSL2 is involved in the fruitlet abscission. In addition, LcHSL2 was demonstrated to play a role in abscission since ectopic expression of *LcHSL2* in Arabidopsis *hae hsl2* mutants background could rescue the floral organ abscission deficiency (Figure 2). However, whether LcIDL1 and LcHSL2 could form a ligand-receptor module in litchi to control the fruit abscission requires further study. Recent studies have showed that IDA-HAE/HSL2 signaling components were found across the plant kingdom [16,29]. Based on these findings, we propose that different cell separation processes might share the IDA-HAE/HSL2 signaling module that is conserved across plant species.

The interaction between the IDA-HAE/HSL2 module and ethylene signaling that functions in the abscission needs further clarification. In the model plant Arabidopsis, *ida* mutants share similar ethylene sensitivity to wild type Arabidopsis, and the deficiency in floral organ abscission of *ida* is unaffected by the exposure of exogenous ethylene [10]. In addition, the *AtHAE* promoter is expressed specifically in floral AZ and its expression does not differ between the ethylene-insensitive mutant *etr1-1* and wild type [12], leading to the conclusion that the IDA-HAE/HSL2 signaling module acts independently of ethylene in regulation of abscission. Recently, a review which reevaluates the relation between ethylene and the IDA–HAE–HSL2 pathway, proposes that the IDA–HAE–HSL2 pathway is essential for the final stages of organ abscission, while ethylene plays a critical role in its initiation and progression [30]. In cultivated crops, ethylene activated the abscission-specific expression of soybean and tomato *IDA*-like genes, and application of ethylene inhibitor repressed the soybean *IDA*-like gene expression [31]. Additionally, recent reports revealed that ethylene activated the expression of *HSL* and *IDA* genes in the AZ of oil palm fruits [16], as well as in the AZ of lupine flowers [32]. In all the above studies, the *IDA* or *HSL* in the AZ were induced prior to the onset of abscission, indicating that the IDA-HAE/HSL2 signaling module is promoted by ethylene, and probably controls the abscission processes downstream of ethylene. In our study, we also found that *LcHSL2* was increased during the ethephon (ETH)-induced fruitlet abscission in litchi (Figure 5). Our findings in this study, and in combination with a previous report for LcIDL1 [21], further support that the IDA-HAE/HSL2 module acts downstream of ethylene signaling in control of abscission. Therefore, it is of great interest in the future to determine which key components involved in the ethylene signaling pathway interact directly with the IDA-HAE/HSL2 module.

## 4. Materials and Methods

### 4.1. Plant Materials and Treatments

For litchi, three 16-year-old litchi trees (*Litchi chinensis* Sonn. cv. Feizixiao) grown in an orchard located in South China Agricultural University (Guangzhou, China) were selected randomly. Similar diameter shoots (about 5–8 mm) bearing 30 fruits and growing in different directions from each tree were tagged. ETH treatments, calculation of cumulative fruit abscission rate (CFAR), and collection of AZ tissues is previously described in [21].

For Arabidopsis to express *LcHSL2* in *hae hsl2* mutants (which are totally deficient in floral organ abscission [5]), the full-length open reading frame of *LcHSL2* was subcloned into the vector pCAMBIA1302 under the control of the *3*5S promoter to generate *35S:LcHSL2* constructs using ClonExpress^®^II One Step Cloning Kit (Vazyme, Nanjing, China). Then, *35S:LcHSL2* constructs were transformed into *hae hsl2* plants following the floral dip method [33]. T1 transgenic lines were used for phenotypic analysis. All the Arabidopsis plants were grown at 22 °C under long day (16 h light/8 h dark) conditions. To reduce variation, all genotypes tested in each experiment were grown together. Primers used here are listed in Table S1.

### 4.2. Quantitative RT-PCR Analysis

Total RNA was isolated from litchi AZ tissues or Arabidopsis leaves (20 days old) using 1 mL Trizol reagent (Invitrogen, Carlsbad, CA, USA). The first strand cDNA synthesis was generated using 2 μg total RNA according to the manual of the TransScript One-Step gDNA Removal and cDNA Synthesis SuperMix Kit (TransGen, Beijing, China). Quantitative RT-PCR analysis was performed as previously described [21]. Primers used here are listed in Table S1.

### 4.3. Subcellular Localization Analysis

The coding sequence of *LcHSL2* was fused into a pBI121 vector that was tagged with GFP to generate 3*5S:LcHSL2-GFP* constructs. Then, *35S:LcHSL2-GFP* constructs were delivered into *Agrobacterium tumefaciens* strain EHA105, and were transformed into tobacco (*Nicotiana benthamiana*) leaves as previously described [34]. YFP and GFP fluorescence were observed with a confocal laser scanning microscope (LSM 7 DUO, ZEISS, Oberkochen, Germany). Primers used here are listed in Table S1.

### 4.4. BCECF Fluorescence Assay

BCECF fluorescence analysis was conducted as described previously [21]. In brief, inflorescences were cut from the plant body and immersed in 10 μM BCECF-AM (B1150, Invitrogen, Carlsbad, CA, USA) solution under darkness for 20 min. The inflorescences were then rinsed four times with phosphate-buffered saline (PBS, pH 7.4) to remove excess BCECE-AM. Images were snapped with a confocal laser scanning microscope (LSM 7 DUO, ZEISS, Germany). Samples were excited by both 488 nm and 633 nm light, then BCECF fluorescence and chlorophyll autofluorescence were detected through 494–598 and 647–721 filters, respectively.

### 4.5. Histochemical GUS Assays

The *LcHSL2* promoter region (−1 to −2000 bp) was subcloned into the vector pCAMBIA1391 to generate the *LcHSL2pro:GUS* constructs. The *LcHSL2pro:GUS* constructs were delivered into Arabidopsis as mentioned above. T1 transgenic plants were used for GUS assays. Transgenic flowers were stained in GUS solution (10 mM EDTA, 0.1% Triton X-100, 2 mM potassium ferricyanide, 2 mM potassium ferrocyanide, 100 μg mL^−1^ chloramphenicol, and 1 mg mL^−1^ X-Gluc in a 50 mM sodium phosphate buffer, pH 7.0) overnight at 37 °C and cleared in a 20% lactic acid/20% glycerol solution for 6 h at 37 °C and then cleared in 70% ethanol. GUS expression was visualized using a Zeiss SV11 stereoscope. Primers used here are listed in Table S1.

## Supplementary Materials

Supplementary materials can be found at https://www.mdpi.com/1422-0067/20/23/5945/s1.
