# Supplementary material for: Identification and Characterization of HAESA-Like Genes Involved in the Fruitlet Abscission in Litchi
Source: Int J Mol Sci. 2019 Nov 26;20(23):5945. doi: 10.3390/ijms20235945 (PMC6928639; doi:10.3390/ijms20235945)
Supplement: Supplementary file 1 [file ijms-20-05945-s001.zip › File S1.pdf]

**>LcHAE (ID number: LITCHI029130.m1)**

MLVFLLCLILSSPPLLTLSLNQDGLLLQRVKLGLSDPTQSLSSWDNNSRDETPCNWQGVT

CDSLSRRVVAVNLDHFQLSGPFPLFFCRLPYLTDLSMFDNSINSTLPLAISTCRNLTYLN

LASNLLVGTVPASLAEMPNLRILDLSANNFSGDIPARFGEVSQLEFLNLAGNLLNGKIPG

WLGNFSSLKELDLAYNPFTPGPIPSELGNMSNLEHLWLAQCNLVGQIPESLTRLNKLINL

DLAINRLSGSIPSSLTELQSIEQIELYQNSFSGELPVKWLNTKTLRRFDASTNHFTGTIP

HELCELQLGSLNLFENRLEGTIPDTIAQSKNLYELKLFNNNLHGELPSQLGKNSPLTALD

VSYNQLTGGIPNGLCSKGSLEDLILIHNSFSGQIPESLGRCQSLRRVRLKQNLLSGSVPE

GLWGVPHMYLFELAENSFTGSIPRRISGAYNLSMFLISKNQFSGSIPDEVGGLGNLVEFS

ASENRFTGRIPGNLVHLDQLGKLDLSQNELSGAMPQGIQRWRNLNELSLANNRLSGEIPS

EIGSLPVLNYLDLSGNLFSGKIPLELQNLKLNLLNLSNNKLSGQLPPLYAKEIYRNSFVG

NPGLCGDLADLCPETGVSKSQGYMWVLRSIFVLAAVVFIVGVVWFYVKFRSFKKTKKGIA

ISKWKSFHRIGFSEFDVIDCLKEENLIGSGASGKVYKAVLRNGEAVAVKKLFERSKKDDT

SNGSLKDQFEAEVETLGKIRHKNIVRLWCCCSNGESKLLVYEYMPNGSLGDLLHSSKAGL

LDWPTRYKIALDAAEGLSYLHHDCVPPIVHRDVKSNNILLDAEFSARVADFGVAKVVDGL

NKGQESMSVIAGSCGYIAPEYAYTLRVNEKSDIYSFGVVLLELVTGKLPIDPEFGEKDLV

KWVCTNLDQKGFDSVIDPKLDSTYKEEICRVLEISLLCTSALPINRPSMRKVVKLLQEAS

ADIKSKITNKKDGKLSPYYYEYTSDQFVV

**>LcHSL1 (ID number: LITCHI029130.m1)**

MVFLYVLILFLFSPLPSLSLNQEGLYLQRVKHTLSDPNSALSSWDNDRDDTPCSWFGVKC

DPRGQSVTSIDLSNANIAGPFPSFVCRLQHLTFLSLYNNSINSTLPDDLATCRNLEHLDL

AQNLLTGTLPDSLADLPSLKYLDLTANNFSGEIPDSFGRFQKLEVISLVYNLLDGTIPPF

LGNISSLRMLNLSYNPFLPGRIPAELGYLTNLEILWLTECNLVGEIPDSLGRLGKLVDLD

LAVNNLGGTIPSSLSGLTSVVQIELYNNSLTGELPTGWSNLTKLRLLDASMNDLSGPIPD

ELTRLPLESLNLYENRFEGDLPESIADSPGLYELRLFRNRLTGQLPKNLGKNSPLKWLDV

SNNQFTGEIPENLCEKGELEELLIIYNSFTGPIPESLGRCQSLTRVRLGYNQLSGEVPPL

FWGLPHVYLLELIDNSFSGQIAKTIAGAANLSLLIISKNNFSGSLPEEIGFLKTLFTFSG

SENRFSGPLPESLKNLGELGTLDLHDNELSGELPTSVRSWKKLNELNLANNEFYGSIPDD

IGTLSVLNYLDLSNNRLSGKIPVGLQNLKLNRLNVSNNRLSGELPPLFAKDMYKSSFIGN

PGLCGDLEGLCGGREGEKNRGYVWLLRSIFVLAAVVFVFGLVWFYLKYRKFKNARAIDRS

KWTLMSFHKLGFSEYEILDGLDEDNVIGSGASGKVYKVVLSNGEAVAVKKLWGGVKKECE

SGDLEKGQAQVQVQDDGFQAEIETLGKIRHKNIVKLWCCCTTRDCKLLVYEYMPNGSLGD

LLHSSKGGLLDWPTRYKIIVDAAEGLSYLHHDCVPPIVHRDVKSNNILLDGDFGARVADF

GVAKVVETSGKPKSMSVIAGSCGYIAPEYAYTLRVNEKSDIYSFGVVILELVTGRRPIDP
EFGERDLVRWVCTTLDQKGVDHVIDSKLDSCFKEEICKVLNIGLLCTSPLPINRPAMRRV
VKLLQEVGAENQPKPAKKDGKLSPYYYEDASDHGSVA

**>LcHSL2 (ID number: LITCHI007137.m1)**

MKYQSIKLFLSLLFSLLYSFSPVNSLKEVTQILFLVKSGQLHDSNGKLNDWVPTSDKSPC
NWTGITCDAQNQSVVGVDLSGLRISGGFPNGFCRIRTLKNLTLTDNSFNGTLSPQSLSPC
SHLQVLELSSNYFTGELPDLPREFSSLQVLNLYNNELSGDIPTSFGRFPALKVLNLGGNY
IGGSIPWFLSNLSELTTLQLGYNPFKPSPLPPSYGNLSKLETLWVARANLVGEIPESIGK
LATLTNLDLSDNFLSGKIPYTIGGLTTVVQIELYYNSLSGELPESLANLTALLRLDVSQN
NLTGTLSQNIASMSLQSLNLNDNHFTGEIPEILALNPNLSELKLFNNSFSGKLPENLGKF
SDLQDFDVSTNDFTGELPPFLCHRNLLQNLVIFSNRFSGNLPKSYGECQPLYYVRMGNNR
LSGEVPAKFWGLPRLEFLEMYNNNFDGPISPSISRARNLTHLLISGNNFSGRIPSAVCDL
HQLLFFNTGRNRLSGEVPSCIAQLNKLQKLDLNQNMFTGELPGRLDLLTDLTELNLSNNR
FTGEIPRELGNLPVLEHLDLSSNMLTGEIPVELTKLKLNQFNVSNNQLYGKVPSGFDHLL
FVSSLLGNLALCSRDLKPLPPCPRNRPETVFFASILAICVVLTLGSVVWFIKTKSNYVNK
PKGRWKVTTFQRVGFTEEDIIPHLTEENLIGSGGSGQVYRVKLKTGQTVAVKRLWSGKLK
LETETVFNSEIEILGRIRHGNIVKLLQCCSGEECRILVYEYMENGSLGDVLHGERGGASL
QDWRTRLTIALGIAQGLAYLHHDCVPTIVHRDVKSNNILLDAEMMPRLADFGLAKTLEAD
GSMSRLAGSYGYIAPEYAYTSKVNEKSDVYSFGVLLMELIMGRRPNDACFGENKDIVRWV
TETALSCPERETTENDSSYCKDLRKLVDPRIKQSTCNYEEIEKVLKVALLCTASFPFNRP
SMRRVVALLNAKAVTRPK
